# Effects of arabinoxylan and chlorogenic acid on the intestinal microbiota in dextran sulfate sodium–treated mice

**DOI:** 10.3389/fnut.2022.950446

**Published:** 2022-11-28

**Authors:** Minhao Xie, Xianzhu Zhang, Xiaoxiao Wang, Guijie Chen, Jianhui Liu, Xiaoxiong Zeng, Wenjian Yang

**Affiliations:** ^1^Collaborative Innovation Center for Modern Grain Circulation and Safety, College of Food Science and Engineering, Nanjing University of Finance and Economics, Nanjing, China; ^2^College of Food Science and Technology, Nanjing Agricultural University, Nanjing, China

**Keywords:** arabinoxylan, chlorogenic acid, intestinal microbiota, dextran sulfate sodium-treated mice, synergistic

## Abstract

Dietary non-starch polysaccharides and phenolics are usually ingested at the same time. They are both regarded as prebiotics, and they regulate the intestinal microbiota through various mechanisms. Notably, however, reports of their combined or synergistic effects are rare. Arabinoxylan (AX), a polysaccharide, and chlorogenic acid (CA), a polyphenol, are widely consumed, and their effects on the microbiota have previously been discussed. In the present study, they were given to dextran sulfate sodium (DSS)–treated mice, separately and together, and the intestinal microbiota were investigated by high-throughput sequencing. The data showed that CA attenuated body weight loss, colon shortening, and histological damage in DSS-treated mice, while neither AX nor the AX+CA combination exhibited any ameliorating potential. AX+CA had less of a modulating effect on intestinal microbiota profiles than did CA. AX+CA administration increased the relative abundance of *Flavonifractor, Coprobacillus*, and *Clostridium_XlVa*, and decreased the abundance of *Robinsoniella* and *Lactobacillus*. Compared to AX and CA, AX+CA contributed to a more complicated shift in the biological functions of the intestinal microbiotaAX seemed to weaken the beneficial effects of CA, at least in the present experimental model of DSS-induced colitis. The combined effects and mechanisms of dietary polysaccharides and phenolic compounds on the intestinal microbiota and on overall health still need to be further investigated.

## Introduction

Low intake of health-benefiting foods, such as whole grains, vegetables, and fruits, has been a major dietary risk factor for mortality and disability-adjusted life-years over the past few decades (1). The beneficial effects of whole grains, vegetables, fruits, and other plant-based foods, including their antioxidant and anti-inflammatory properties, as well as the attenuation of metabolic disorders, could come from their functional ingredients, such as non-starch polysaccharides, phenolics, and other bioactive components (2). Arabinoxylan (AX) is the main non-starch polysaccharide in cereal grains, including maize, rye, barley, oats, sorghum, wheat, rice, and other plants. It accounts for between 11 and 26% of wheat bran and between 60% and 70% of the endosperm cell wall and aleurone layer (3). The main chain structure of AX consists of a β-1,4-D-Xyl*p* backbone with L-Ara*f*. AX exhibits many bioactive characteristics, including antioxidant, fermentable, and prebiotic properties, glucose- and lipid-regulating effects, and immune-modulating potentials (4, 5). AX has been described as an efficient immunomodulator for synergistic or complementary cancer treatment (6). Chlorogenic acid (CA), a hydroxycinnamic acid–derived phenolic compound, is widely distributed in many plant foods, including vegetables, fruits, and herbal remedies (7). It is an extensively studied phytochemical that possesses many health-promoting properties, such as antioxidant, anti-inflammatory, antidiabetic, antilipidemic, and antihypertensive activities (8).

Inflammatory bowel disease (IBD) has become a global disease and health burden with increasing incidence. Although its exact aetiopathogenesis remains unclear, it is widely known that the gut microbiota is closely associated with IBD. Some members of the gut microbiome and bacterial metabolites, including *Faecalibacterium prausnitzii, Roseburia*, short-chain fatty acids, and secondary bile acids, have potentially protective effects against IBD, while others, such as adherent invasive *Escherichia coli, Enterococcus faecium*, enterotoxigenic *Bacteroides fragilis*, and *Campylobacter concisus*, show potentially causative effects (9). Diet also plays an important role in the development and progression of IBD. Epidemiological studies have identified potential dietary risk factors for IBD, such as red meat, processed foods, refined sugar, and saturated fat, and dietary intervention studies have shown promising results, demonstrating that diets rich in fruits, vegetables, whole grains, and seafood are associated with reduced risk (10).

Dietary fermentable polysaccharides and phenolics have modulating effects on the intestinal microbiota, and they are considered prebiotics (11). In *in vitro* studies of fecal samples, AX exhibited significant effects on the microbiota, including increased abundance of *Collinsella, Blautia*, and *Bifidobacterium*, and decreased abundance of *Sutterella, Bilophila*, and *Parabacteroides*. It also significantly increased the amount of total and individual short-chain fatty acids (SCFAs) (12). AX has also been shown to contribute to a global shift in the intestinal microbiota profile in overweight or obese adults, promote the growth of operational taxonomic units related to *Bifidobacterium longum, Blautia obeum*, and *Prevotella copri*, and increase the concentration of fecal propionate (13). Supplementation of AX promoted the proliferation of fiber-degrading bacteria and the generation of short-chain fatty acids (SCFAs), and decreased the abundance of opportunistic pathogens, in type 2 diabetic mice (14). Phenolic CA has been shown to increase the relative abundance of SCFA-producing bacteria, including *Bacteroides, Prevotellaceae UGC-001*, and *Butyricimonas*, in hyperuricemic mice (15). It also promoted the abundance of *Bifidobacterium* and decreased the content of *Escherichia coli* in the feces of mice with non-alcoholic fatty liver disease (16).

Dietary bioactive polysaccharides and phenolics both have prebiotic effects on the intestinal microbiota, and they exert regulating potential *via* distinct mechanisms. However, there has been little research into their combined effects. The present study investigated the effects of AX, CA, and their combination on the intestinal microbiota of mice treated with dextran sulfate sodium (DSS). Our data have the potential to provide novel insights into the synergistic potential of dietary components.

## Materials and methods

### Study materials and animal diet

AX (derived from wheat, low viscosity, Lot 160419b) was purchased from Megazyme Ltd. (Bray, Ireland). CA was obtained from Aladdin Biochemical Technology Co., Ltd. (Shanghai, China). Colitis-grade DSS was obtained from MP Biomedicals (Irvine, CA). AIN-93G purified rodent diet was purchased from Jiangsu Xietong Pharmaceutical Bio-engineering Co., Ltd. (Nanjing, China).

### Experimental procedure

**Figure 1:**
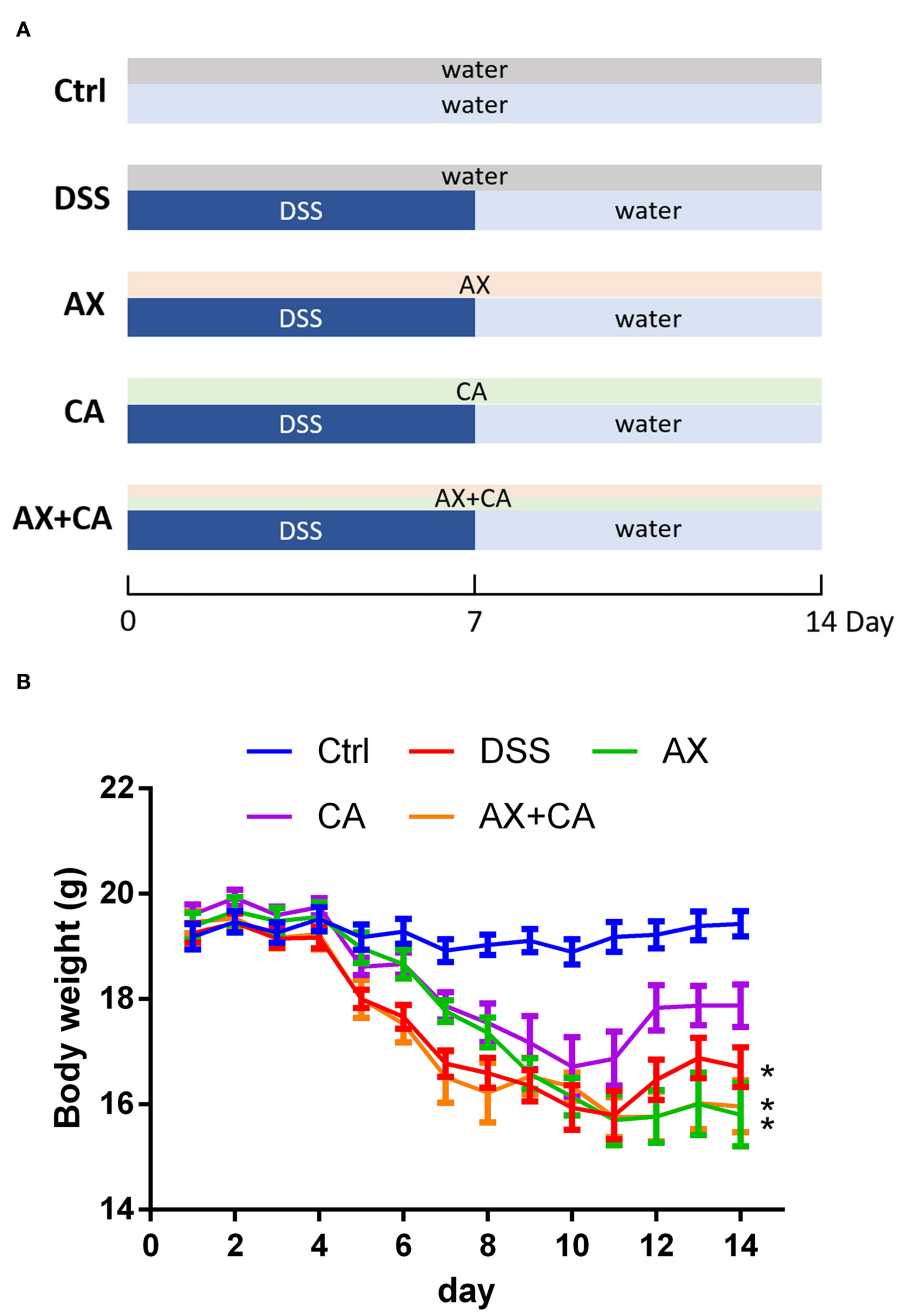
**(A)** Experimental design and treatment structure; **(B)** Changes in body weight over time for each group. The * symbol indicates the body weight of mice in the group was significantly different from those in the Ctrl group after feeding and treatment for 14 days (*p* < 0.05).

The animal experimental protocol was approved by the Ethical Committee of Nanjing Agricultural University Animal Experiment Center (SYXK- <Jiangsu>-2021-0086) according to the National Guidelines for Experimental Animal Welfare. Forty 6-week-old C57BL/6 mice were kept in a specific pathogen-free facility under standard laboratory conditions. After acclimating for 1 week, they were randomly divided into five groups. The feeding and treatment procedure are illustrated in Figure 1A. The mice in the normal control (Ctrl) and DSS-treated (DSS) groups were daily orally gavaged with water. The mice in the AX and CA groups were daily given AX and CA, respectively, at a dose of 200 mg/kg body weight. The mice in the AX+CA group were administered a combination of AX and CA dosed at 200 mg/kg body weight. The mice in the Ctrl group freely drank water. The other mice had free access to DSS solution (1.5%, w/v) for the first 7 days, then the DSS solution was changed to water for the remaining days. After 14 days of treatment, fresh feces were collected, immediately frozen in liquid nitrogen, and stored at −80°C until further analysis.

### Histological analysis of colon tissue

After sacrifice, the distal colon tissues of the mice were immediately fixed with 4% formalin, and they were washed, dehydrated, embedded into paraffin, sliced into 5 μm sections, and transferred onto glass slides. The slides were stained with hematoxylin-eosin reagent (Sigma-Aldrich, St. Louis, MO), and a histological examination was performed, with a focus on crypt architecture, inflammatory cell infiltration, goblet cell depletion, and muscle tissue thickening (17).

### Gut microbiota analysis

The high-throughput amplicon sequencing and gut microbiota analysis were performed by Genesky Biotechnologies Inc. (Shanghai, China) according to a general pipeline. The total DNA of the fecal samples was extracted with the QIAamp Fast DNA Stool Mini Kit (Qiagen, Germany). The resulting DNA was amplified with barcoded specific bacterial primers targeting the variable region 3 (V3) and V4 of the 16S rRNA gene using Primer F (5'-Illumina adapter sequence 1+ CCTACGGGNGGCWGCAG-3') and Primer R (5'-Illumina adapter sequence 2+ GACTACHVGGGTATCTAATCC-3'). The sequencing was performed on an Illumina NovaSeq 6000 platform *via* the SP-Xp two-end strategy. An amplicon sequence variants (ASVs) table was generated after the raw sequencing data were filtered, denoised, merged, and depleted of chimeras with the QIIME2 and DAD2 plugins (18, 19). Taxonomic assignments of the ASV sequences were conducted by a naive Bayes classifier with a confidence threshold of 0.8; the classifier had been pretrained on the RDP database (version 11.5). The function of the bacterial community was predicted by Phylogenetic Investigation of Communities by Reconstruction of Unobserved States (PICRUSt2) software (20).

### Statistics

The data were expressed as mean ± standard error of the mean, and statistical significance was tested by one-way analysis of variance (ANOVA). A *p* < 0.05 was considered statistically significant. The difference of microbial taxon was determined by Metastats software and the linear discriminant analysis effect size (LEfSe) package (21, 22).

## Results

### Effects of AX and CA on body weight

Body weight is one of the most important objective indicators of nutrition and health status. Body weight changes in the mice were therefore analyzed to assess whether the treatments had any effects on their body condition. Administration of DSS decreased body weight due to colon inflammation. As shown in Figure 1B, at the experimental endpoint, the body weights of the mice in the DSS, AX, and AX+CA groups were still lower than those of the Ctrl group (*p* < 0.05). However, the body weights of the CA group mice were attenuated, remaining closest to those of the Ctrl group (*p* > 0.05), suggesting the ameliorating effects of CA on colitis. Unfortunately, the combination of AX and CA did not show satisfying potential with regard to body weight.

### Effects of AX and CA on colon length and histological structure

**Figure 2:**
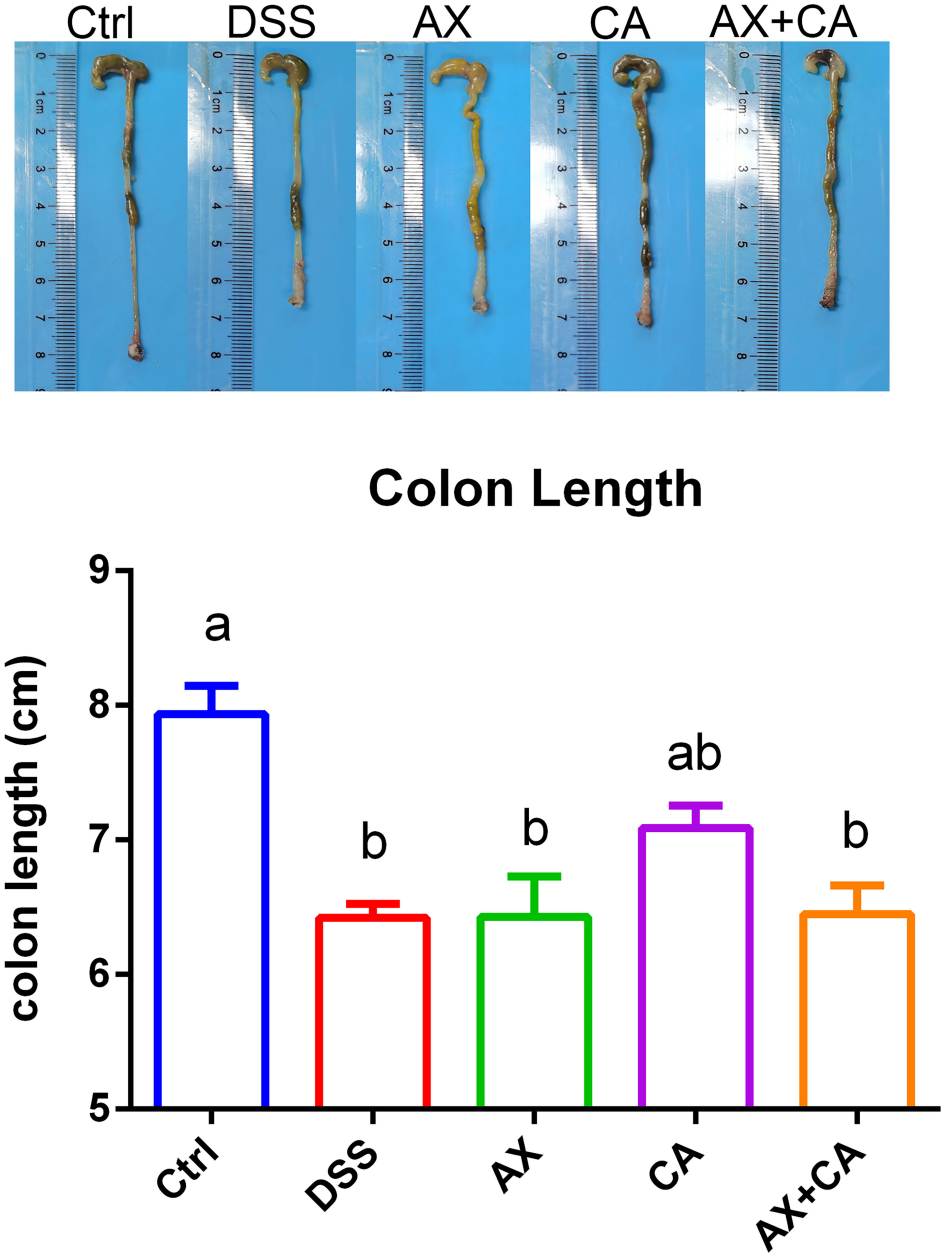
Colon lengths of the mice in each group.

**Figure 3:**
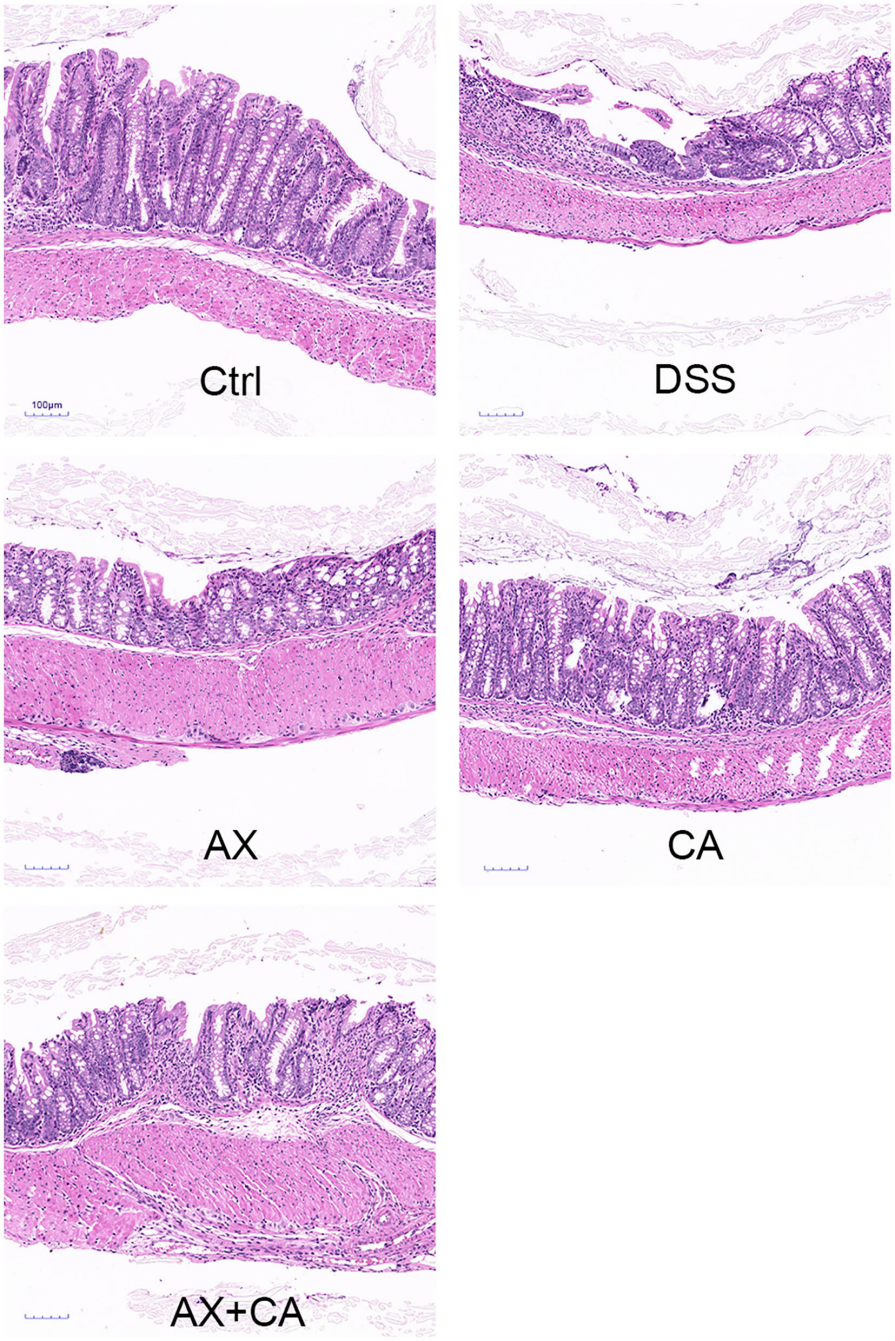
Histological observations of colon tissue from mice in each group.

The colon lengths of the mice in each group are presented in Figure 2. DSS administration significantly shortened colon length, as compared to those of mice in the Ctrl group (*p* < 0.05). CA supplementation reversed these losses, ultimately restoring the colon lengths of CA group mice to lengths similar to those of Ctrl group mice (*p* > 0.05); however, the AX and AX+CA treatments did not show any attenuating potential. As shown by the histological examination (Figure 3), the crypt damage caused by DSS administration was remedied by CA treatment, while AX and AX+CA supplementation had only slight effects on colon crypt structure. This is consistent with the data on body weight and colon length.

### Effects of AX and CA on the diversity and similarity of the intestinal microbiota

The alpha-diversity of the mice's intestinal microbiota was evaluated with the Chao1, Shannon, and Simpson indices (data not shown), but the results did not demonstrate a difference in the diversity of intestinal microbiota across the five groups.

**Figure 4:**
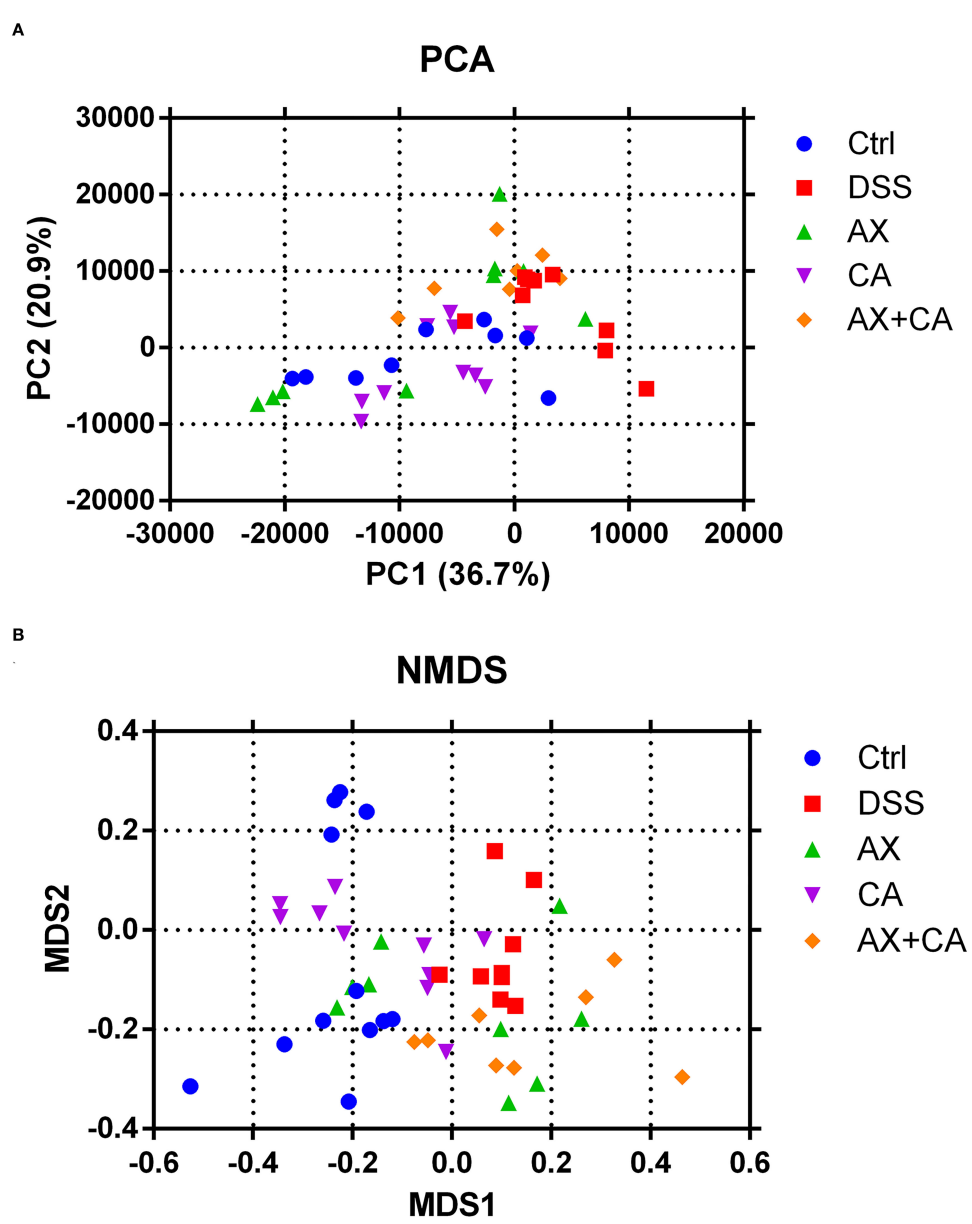
Beta-diversity of intestinal microbiota [**(A)** PCA; **(B)** NMDS].

Beta-diversity is widely used to compare the similarity of different ecosystems. To identify possible differences between the intestinal bacterial profiles among the five groups, as well as the changes in gut microbiota derived from the AX and CA interventions, the beta-diversity of the samples was assessed using principal coordinates analysis (PCA) and non-metric multidimensional scaling (NMDS). The results are displayed in Figure 4.

The results derived from different methods exhibited similar tendencies, and the data showed that the intestinal microbial profiles of DSS group mice were significantly different from those in the Ctrl group. Intervention with AX and CA shifted the overall intestinal microbial profiles in DSS-treated mice toward those of the Ctrl group, suggesting their modulating effects on the intestinal microbiota. The results further indicated that the microbial profile of CA group mice was closest that of Ctrl group mice, while AX+CA supplementation had little effect on the beta-diversity of the intestinal microbiota. These results were consistent with the tendencies illustrated by the findings for body weight and colon morphological structure.

### Effects of AX and CA on microbial compositions

**Figure 5:**
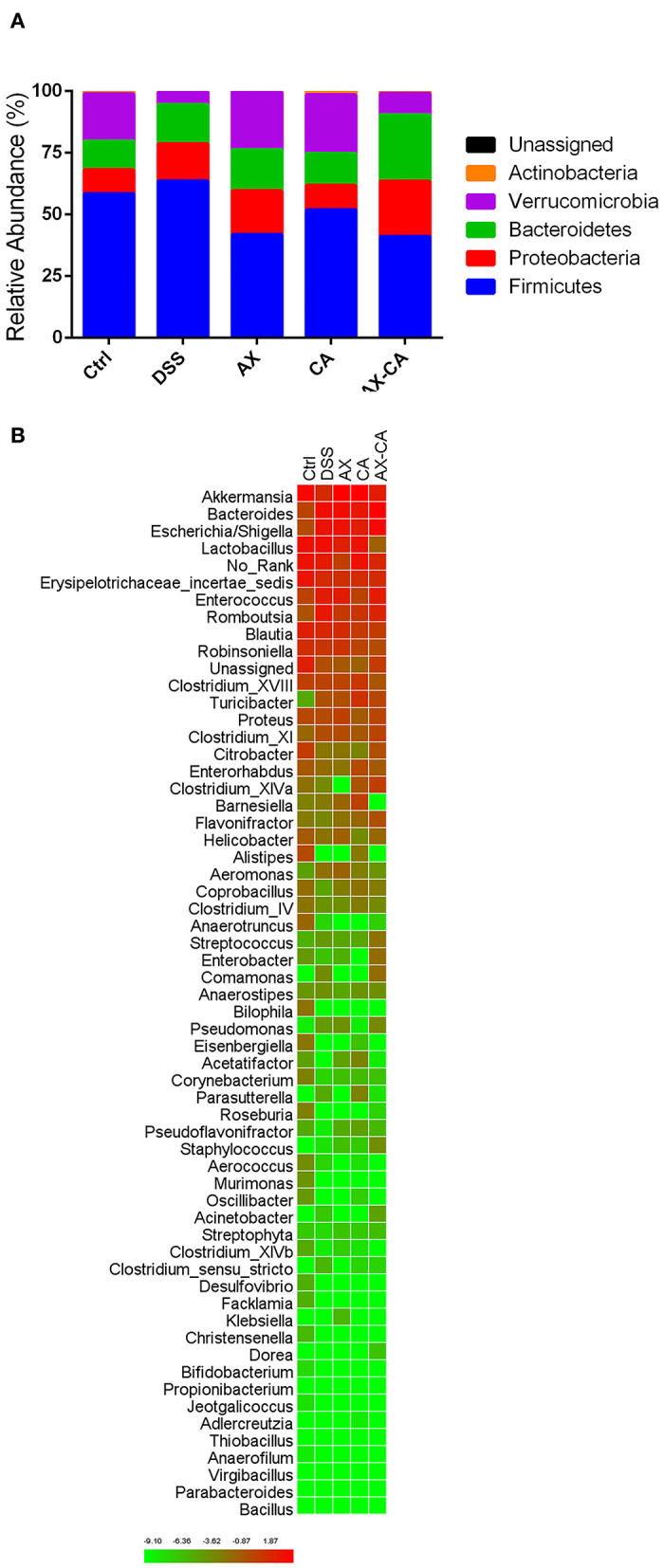
Intestinal microbial composition of the mice in each group, at the phylum **(A)** and genus **(B)** levels.

The taxonomic compositions of the intestinal microbiota of each group, at both the phylum and genus levels, are displayed in Figure 5. At the phylum level, *Firmicutes, Proteobacteria, Bacteroidetes, Verrucomicrobia*, and *Actinobacteria* were the dominant communities. DSS treatment significantly decreased the relative abundance of *Verrucomicrobia* and *Actinobacteria*. AX and CA supplementation both increased the relative abundance of *Verrucomicrobia* and decreased the level of *Firmicutes*. Notably, the combined administration of AX+CA did not significantly affect the relative abundance of *Verrucomicrobia* or *Actinobacteria*. At the genus level, *Lactobacillus, Akkermansia, Bacteroides, Escherichia/Shigella, Romboutsia, Enterococcus, Erysipelotrichaceae incertae sedis, Blautia, Parasutterella, Robinsoniella, Turicibacter, Clostridium* XVIII, *Proteus, Clostridium* XI, *Enterorhabdus, Bifidobacterium, Barnesiella, Citrobacter*, and *Streptococcus* were the most abundant communities.

**Figure 6:**
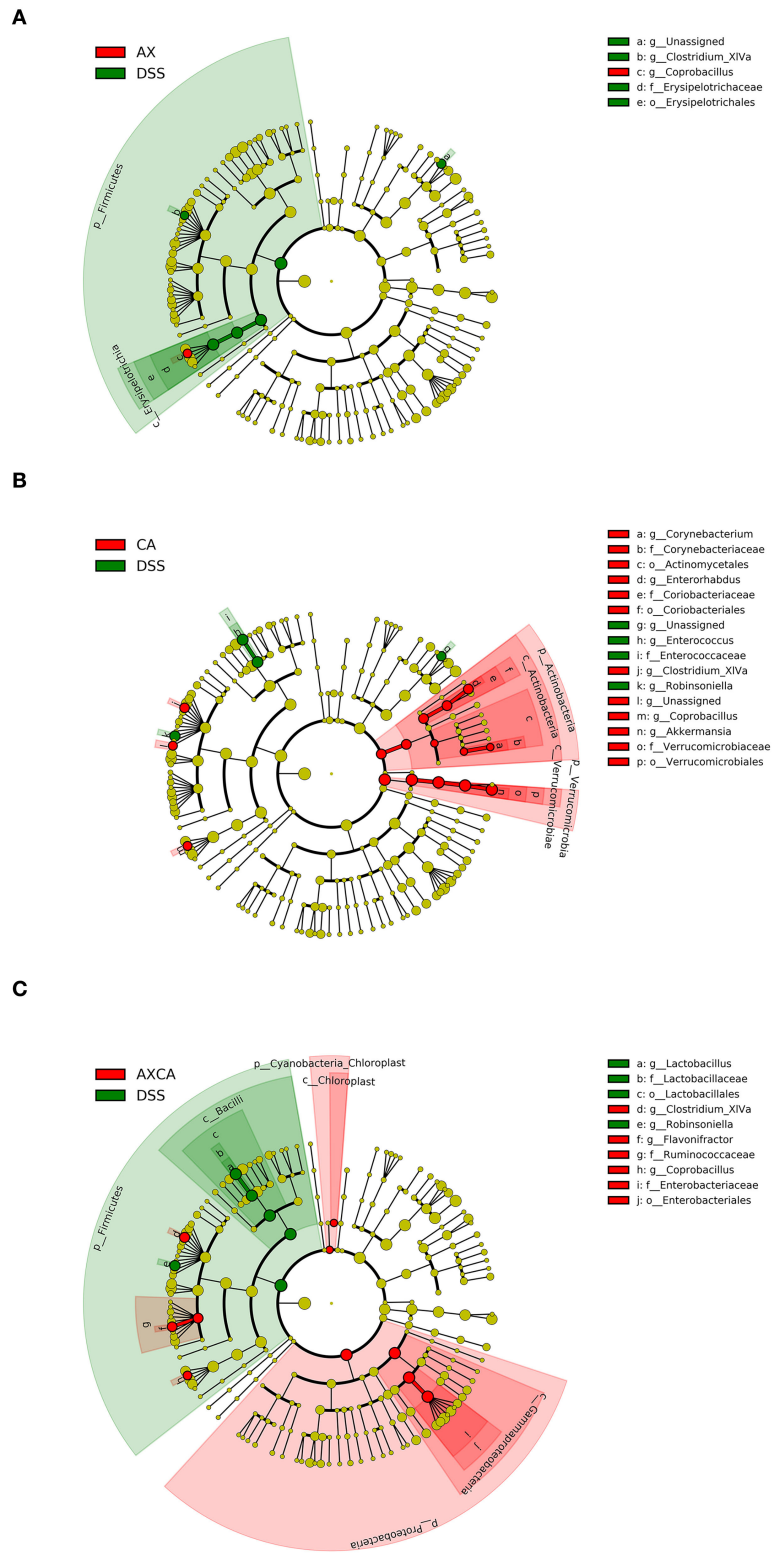
LEfSe cladogram plot of the intestinal microbiota of mice in the AX **(A)**, CA **(B)**, and AX+CA **(C)** groups, compared to those in the DSS group.

As shown in Figure 6, LEfSe was used for discriminant analysis of multilevel species differences. For each group, bar charts were drawn for the highest relative abundance. Bars with different colors represent the different species in different groups with an LDA score > 2. Compared to the DSS group, CA supplementation decreased the relative abundance of *Enterococcus* and *Robinsoniella* and increased the relative abundance of *Coprobacillus, Clostridium* XIVa, *Enterorhabdus, Corynebacterium*, and *Akkermansia* at the genus level. AX decreased the relative abundance of *Clostridium* XIVa and increased the level of *Akkermansia* and *Coprobacillus*. The combination of AX+CA decreased the relative abundance of *Lactobacillus* and *Robinsoniella* and increased the relative abundance of *Coprobacillus, Flavonifractor*, and *Clostridium* XIVa. Interestingly, AX and CA exhibited similar regulating effects on certain communities, such as *Coprobacillus*, and opposite effects on other communities, such as *Clostridium* XIVa. In addition, AX+CA supplementation significantly modulated the levels of some communities, including *Lactobacillus* and *Flavonifractor*, and they were not significantly affected by solo AX or CA.

### Effects of AX and CA on the biological functions of the intestinal microbiota

**Figure 7:**
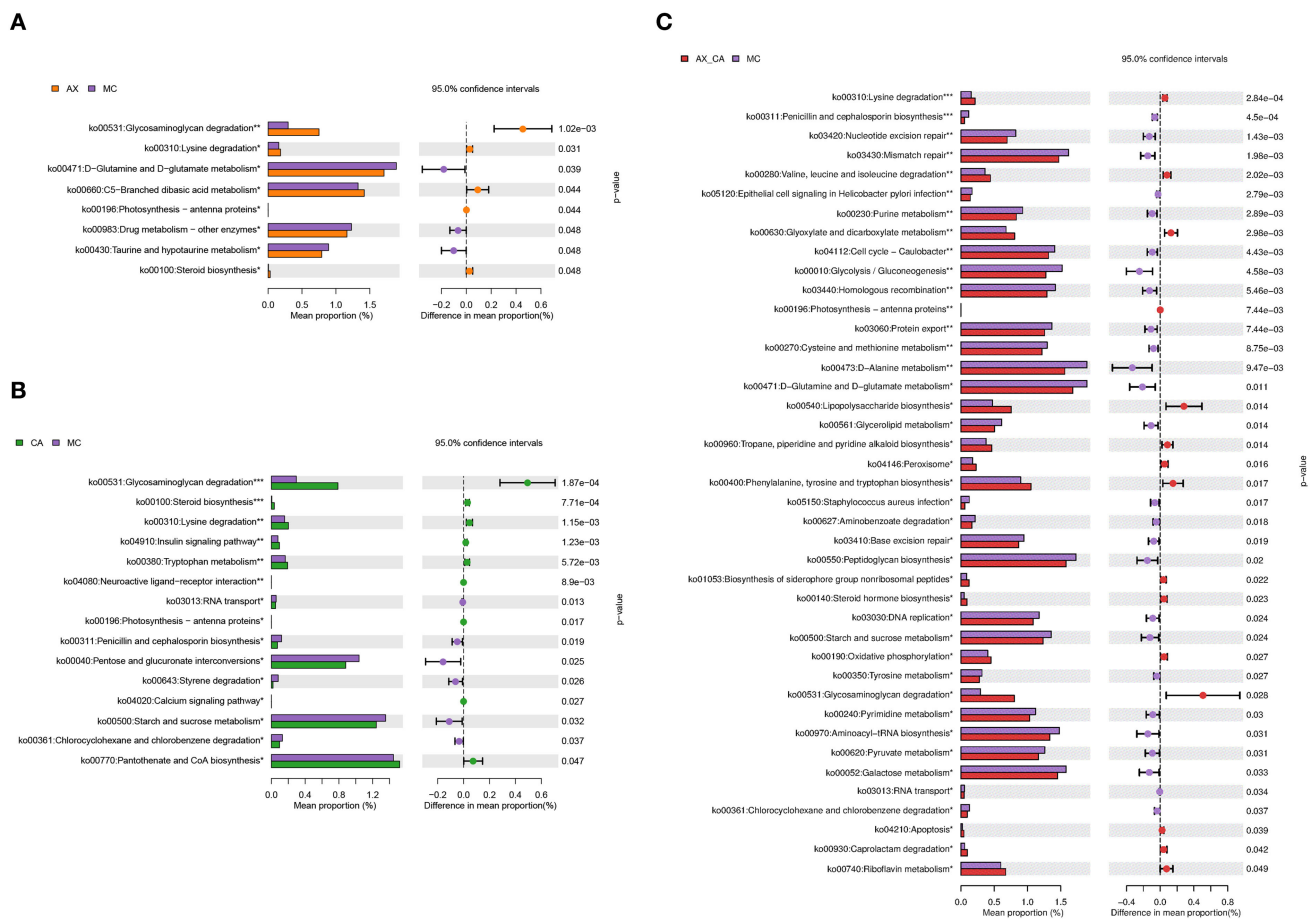
Biological functions according to the differences in intestinal microbiota of mice in the AX **(A)**, CA **(B)**, and AX+CA **(C)** groups, compared to those of the DSS group.

As shown in Figure 7, the combination of AX and CA significantly regulated the biological functions of the microbial communities. Compared to those of the DSS group, supplementation with AX or CA alone mainly contributed to changes in carbohydrate and amino acid metabolism. However, the modulation of microbial functions derived from the combination of AX+CA was much more sophisticated. AX+CA supplementation had a significant impact on nitrogen metabolism, carbohydrate metabolism, and cellular components, including amino acids, nucleotide bases, monosaccharides, lipids, and lipopolysaccharide biosynthesis.

## Discussion

Dietary non-starch polysaccharides and phenolics are usually ingested at the same time. They are both considered prebiotics, and they regulate intestinal microbiota through various mechanisms. The exact mechanisms regarding the modulation of intestinal microbiota by polyphenols remain unclear, though it is believed that they could exert regulating potential through antibacterial effects derived from phenol groups. Phenolic compounds can interact with bacterial development as well as substance and energy metabolism, and interfere with cellular membrane integrity and function. In addition, phenolics could inhibit bacterial biofilm formation and quorum sensing (23). On the other hand, non-digestible carbohydrates can modulate the composition and function of the intestinal microbiota as energy sources for some microbial communities. These microorganisms encode certain glycosidases and are capable of degrading and utilizing the given carbohydrates (24). However, their combined or synergistic effects have rarely been discussed in the literature.

Arabinoxylan, a polysaccharide, and chlorogenic acid, a polyphenol, are widely consumed, and their effects on the microbiota have been described in the literature. In the present study, they were given to DSS-treated mice, separately and together, and the intestinal microbiota were investigated by high-throughput sequencing. The data provided insights into the combined effects of different prebiotics.

AX has been shown to promote the proliferation of *Prevotella 9, Megamonas*, and *Bifidobacterium* in fermentation by pig or duck intestinal microbiota (25, 26). It has also been observed to increase *Lactobacillus, Bifidobacterium*, and *Bacteroidetes* populations, and reduce *Escherichia coli* populations, in the colonic digesta of high-fat diet–fed mice (27). AX-containing diets promoted probiotic *Lactobacillus* and *Bifidobacterium* populations, compared to a basal control diet without fiber, in piglets (28). Supplementation with AX oligosaccharides promoted the growth of bifidobacteria in the cecum of mice fed on a high-fat diet (29). Previous research into the effects of AX on the intestinal microbiota mainly focuses on *Bifidobacterium, Lactobacillus*, and *Bacteroidetes*. These populations are known widely as probiotics; their genomes encode glycoside hydrolase family genes, including xylanases and arabinofuranosidases (3, 30). AX-containing diets have been found to downregulate the gene expression of pro-inflammatory cytokines (tumor necrosis factor [TNF]-α, interleukin [IL]-1β, and IL-6) and the TLRs/MyD88/NF-κB pathway, compared to a basal control diet without fiber, in piglets (28). Wheat bran–derived AX oligosaccharides also reduced metabolic endotoxemia, macrophage infiltration in the adipose tissue, and plasma IL-6 in high-fat diet–fed mice, and improved tight junction proteins (ZO-1 and claudin 3) and gut barrier function (29). A synbiotic composed of AX and *L. fermentum* HFY06 significantly reversed histopathological changes in the colon and inhibited the activation of the NF-κB signaling pathway and the expression of TNF-α, iNOS, and COX-2, alleviating DSS-induced colitis (31). However, many of the previous *in vivo* studies of AX were performed in mice fed a basal diet or high-fat diet, and the effects of AX on DSS-treated mice have rarely been reported. It should be noted that the modulation of the intestinal microbiota seen with AX supplementation could be highly individualized (13), and the intestinal microbiota–modulating effects of AX seen in the present study may differ from previous data due to different experimental inducements, feeding conditions, and dietary fiber sources (32).

CA has the potential to regulate the intestinal microbiota under various physiological conditions. CA has been shown to increase the relative abundance of *Akkermansia* and *Bacteroides*, and reduce the populations of *Erysipelatoclostridium, Faecalibaculum*, and *Erysipelotrichaceae*, in L-carnitine–fed mice (33). It has also been found to increase the population of *Lactobacillus* and decrease that of *Escherichia coli* in weaned piglets (34). Pigs fed a CA-containing diet had a higher abundance of *Lactobacillus, Prevotella, Anaerovibrio*, and *Alloprevotella* in the cecum (35). CA administration alleviated gut dysbiosis by enhancing the relative abundances of *Ruminiclostridium 9, Alloprevotella*, and *Rikenella* in cadmium-treated mice (36). CA also increased the relative abundance of *Bacteroides, Prevotellaceae UGC-001*, and *Butyricimonas*, and reversed the purine metabolism and glutamate metabolism functions of the gut microbiota, in hyperuricemic mice, and it inhibited the expression of pro-inflammatory cytokines and activation of the TLR4/MyD88/NF-κB signaling pathway in the kidney (15). It also reduced the growth of *Bacteroides* and *Bacteroides*-derived liposaccharides and increased the abundance of *Lactobacillus* in indomethacin- and DSS-induced inflammation, respectively (37, 38).

CA has been shown to activate the Nrf-2/HO-1 pathway in DSS-treated mice, upregulate the expression of anti-inflammatory cytokines, antioxidant enzymes, and gut-tight junction proteins, and alleviate colitis (39). CA has also been reported to attenuate DSS-induced colitis by promoting the growth of *Akkermansia* in mice (40). Our data are consistent with these previous results.

The combined effects of phenolics and polysaccharides on colon inflammation have only rarely been described in the literature. It was hypothesized that AX and CA, belonging to two different categories of prebiotics, could synthetically modulate the intestinal microbiota to attenuate DSS-induced colitis. Unfortunately, the combination of AX+CA did not exhibit the expected benefits on colon inflammation, at least in the present mice model of DSS-induced colitis; AX depleted the colitis-ameliorating potential of CA. Compared to the DSS group, AX+CA administration increased the relative abundance of *Flavonifractor, Coprobacillus*, and *Clostridium_*XIVa, and decreased the populations of *Robinsoniella* and *Lactobacillus*.Gut-derived *Flavonifractor* species could be involved in degrading flavonoids; oral administration of *F. plautii* has been shown to promote recovery from acute colitis in mice *via* suppression of IL-17 (41), attenuation of inflammatory responses in obese adipose tissue (42), and suppression of Th2 immune responses (43). An association between *F. plautii* and the gut microbiome was identified in patients with colorectal cancer in India (44), and it is regarded as a potential pathogen for cholecystitis (45).

The *Eubacterium*-like genus *Coprobacillus* nov. was first isolated and identified from human feces in 2000 (46). *Coprobacillus* is thought to positively interact with *Akkermansia* and *Blautia*; they maintain intestinal stability and confer resistance against colonization with the pathogen *Clostridium difficile* (47). The abundance of *Coprobacillus cateniformis* was improved by supplementation of chitosan and positively correlated with serum leptin in high-fat diet–fed mice (48). The abundance of *Coprobacillus* was found to be decreased in the gut microbiota of patients with acne (49). Notably, *C. cateniformis* has been reported to cause bacteremia (50). Besides the effects on microbial composition, AX+CA contributed to more complex changes in the biological functions of the intestinal microbiota.

In summary, CA attenuated body weight loss, colon shortening, and histological damage in DSS-treated mice, while AX and the combination of AX+CA did not exhibit any potential to ameliorate these effects. AX+CA had less of a modulating effect on the profile of the intestinal microbiota than did CA. AX+CA administration increased the relative abundance of *Flavonifractor, Coprobacillus*, and *Clostridium* XIVa, and decreased the populations of *Robinsoniella* and *Lactobacillus*, in DSS-treated mice. Compared to AX or CA alone, AX+CA contributed to a more complicated shift in the biological functions of the intestinal microbiotaAX seemed to weaken the beneficial effects of CA, at least in the present experimental model of DSS-induced colitis. The combined effects and mechanisms of dietary polysaccharides and phenolic compounds on the intestinal microbiota and on overall health still need to be investigated further.

## Data availability statement

The original contributions presented in the study were included in the article, and further inquiries can be directed to the corresponding author. The raw sequence data generated and presented in the study were deposited in the Genome Sequence Archive in the BIG Data Center Chinese Academy of Sciences (https://ngdc.cncb.ac.cn/gsa/) with Bio Project number PRJCA013252 and accession number CRA008935.

## Ethics statement

The animal study was reviewed and approved by Ethical Committee of Nanjing Agricultural University Animal Experiment Center.

## Author contributions

MX: conception, investigation, formal analysis, writing—original draft, and funding acquisition. XZh and XW: investigation and formal analysis. GC: investigation and project administration. JL: writing—review and editing. XZe and WY: conception, supervision, writing—review, and funding acquisition. All authors contributed to the manuscript and approved the submitted version.

## Funding

This research was supported by the National Natural Science Foundation of China (32001700), the Natural Science Foundation of Jiangsu Province (BK20221347), and the Priority Academic Program Development of Jiangsu Higher Education Institutions (PAPD).

## Conflict of interest

The authors declare that the research was conducted in the absence of any commercial or financial relationships that could be construed as a potential conflict of interest.

## Publisher's note

All claims expressed in this article are solely those of the authors and do not necessarily represent those of their affiliated organizations, or those of the publisher, the editors and the reviewers. Any product that may be evaluated in this article, or claim that may be made by its manufacturer, is not guaranteed or endorsed by the publisher.
